# Supporting Participants in Preclinical Alzheimer’s Trials: Experience from a Research Participant Advisory Board and the A4 Study

**DOI:** 10.1002/alz.085977

**Published:** 2025-01-09

**Authors:** Sarah Walter, Guadalupe Morales, Elizabeth Shaffer‐Bacareza, Jason Karlawish, Joshua D Grill, Michael C. Donohue, Paula Cohen, Paul S. S Aisen, Reisa A Sperling, The A4 Study Team

**Affiliations:** ^1^ Alzheimer’s Therapeutic Research Institute, University of Southern California, San Diego, CA USA; ^2^ Alzheimer’s Clinical Trials Consortium, Research Participant Advisory Board, San Diego, CA USA; ^3^ University of Pennsylvania, Philadelphia, PA USA; ^4^ Institute for Memory Impairments and Neurological Disorders, University of California, Irvine, Irvine, CA USA; ^5^ Alzheimer’s Therapeutic Research Institute, Keck School of Medicine, University of Southern California, San Diego, CA USA; ^6^ Massachusetts General Hospital, Brigham and Women’s Hospital, Harvard Medical School, Boston, MA USA

## Abstract

Preclinical Alzheimer’s disease (AD) trials can involve multiple years of follow‐up and burdensome procedures for older individuals. Optimizing the design and conduct of these trials requires input from participants and their families. Since 2020, the Alzheimer’s Clinical Trials Consortium (ACTC) Research Participant Advisory Board has provided input on study attributes including: participant and study partner compensation, consent language, and result communication tools. A key recommendation from the advisory board is to design studies with the option to learn individual research results. Participants find results personally meaningful, even when the clinical relevance of results has not been established. After release of topline study results, A4 research sites were provided reports that included treatment arm assignment and individual research results (cognitive scores, amyloid PET tertile, and Clinical Dementia Rating scale results). Research sites were provided guidance on communicating research results in a training webinar. A survey was subsequently sent to all A4 sites to understand what methods were used to communicate results and support participants. The 42 sites that completed the survey reported sharing results with 445 participants, or 65% of participants active at the end of the study. Participants expressed interest in taking part in other studies and, where eligible, were referred. Those with worsening cognition and function were referred for clinical evaluation and care. Some A4 participants told sites that they expected “something tangible” and “more than a one‐hour meeting” after the many years of participation. The A4 sites that hosted ‘thank you’ events to discuss study‐level results, with separate one‐on‐one sessions to discuss individual results shared these were appreciated by participants. The active involvement of the diverse ACTC Participant Advisory Board has benefited the design of preclinical AD studies. The A4 experience demonstrates that it is feasible to share individual research results, and that a majority of participants will opt to learn. Greater involvement of the Advisory Board at the earliest stages of study design and planning will maximize impact of their feedback, and help researchers move toward the goal of optimized, inclusive trials.

